# Adjacent-possible ecological niche: growth of *Lactobacillus* species co-cultured with *Escherichia coli* in a synthetic minimal medium

**DOI:** 10.1038/s41598-017-12894-3

**Published:** 2017-10-16

**Authors:** Kouhei Mizuno, Mamiko Mizuno, Mio Yamauchi, Aya J. Takemura, Veronica Medrano Romero, Kazuya Morikawa

**Affiliations:** 1 Department of Creative Engineering, National Institute of Technology, Kitakyushu College, Kitakyushu, 802-0985 Japan; 2 Graduate School of Comprehensive Human Sciences, University of Tsukuba, Tsukuba, 305-8575 Japan; 3 PhD Program in Human Biology, School of Integrative and Global Majors, University of Tsukuba, Tsukuba, 305-8575 Japan; 4 Division of Biomedical Science, Faculty of Medicine, University of Tsukuba, Tsukuba, 305-8575 Japan

## Abstract

In certain conditions, members of the *Lactobacillus* genus are auxotrophs that have fastidious requirements for growth. Notably, *Lactobacillus* cannot grow in M9 medium, a minimal synthetic medium used for *Escherichia coli*. However, we found that some *Lactobacillus* strains can be grown in M9 when co-cultured with *E. coli* K-12. In the co-culture, *L. casei* proliferates exponentially, reaching cell densities of 10^8^ CFU (colony-forming unit) ml^−1^ in 6 h and dominating *E. coli* in the late growth phase. Spent medium from *E. coli* grown overnight lacked this growth-promoting effect on *L. casei*. Similarly, the effect was not observed when the species were separated by a 0.4-µm membrane. Microscopic observations showed that *L. casei* are embedded in the micro-scale clusters of *E. coli* in the early growth phase. This study describes for the first time the ability of a *Lactobacillus* species to grow in minimal medium when in close proximity with co-cultured bacteria.

## Introduction

*Lactobacillus* is a group of lactic acid bacteria (LAB) that ferment hexose sugars to produce primarily lactic acid. Some species have been widely used in fermented foods and are recognized as health-promoting ingredients (probiotics). Demonstrated probiotic effects include direct antagonism against pathogens^1^, immunomodulatory properties^2^, and indirect effects (via the fermented products) in reducing blood pressure^3^. Some probiotic strains like *L. rhamnosus, L. casei*, and *L. johnsonii* potentially have therapeutic effects on chronic inflammatory bowel diseases^4,5^. Despite such health-promoting and therapeutic relevance, information on the physiology of LAB is still lacking. Indeed, LAB constitutes only a small fraction (<0.1% among microbiota) of the autochthonous colonizers of the human intestine, and continuous intake of probiotics is necessary to sustain transient LAB at high levels (up to several percent of microbiota) in this environment^6–8^.

In certain conditions, members of the *Lactobacillus* genus are auxotrophs^8^. Intensive studies on synthetic media^9–11^ and recent genomic data reveal that lactobacilli often lack the capacity to synthesize amino acids, vitamins, purines, or fatty acids^12–14^. *L. johnsonii, L. acidophilus*, and *L. gasseri*, commonly found in the human gastrointestinal tract, are auxotrophic for all or the majority of physiologically relevant amino acids^15^. The diversity of *Lactobacillus* auxotrophy is assumed to reflect their adaptation to ecological niches such as gastrointestinal tracts, protein-rich foods, and plant materials^14,16^.

It has been shown that lactobacilli employ multiple strategies for ecological adaptation, including amino acid transport^12^, mucus adherence^17–19^, adhesion to Peyer’s patches^20^, and cell surface-associated proteinases^21,22^. Another ecological aspect of *Lactobacillus* auxotrophy is their inter-species interactions with other microbiota. For instance, interaction between LAB and yeast has been observed in fermented foods and has long been a topic of study^23–25^. Auto- and co-aggregation have been reported for various *Lactobacillus* species, including *L. crispatus, L. gasseri, L. reuteri*, and *L. coryniformis* ^26^; these phenomena have been shown to involve several aggregation-promoting factors such as Apf ^27^ and Cpf ^26^. Recently, we reported the co-aggregation of *L. casei* NBRC 3831 with *E. coli* K-12, including the demonstration that *E. coli* fimbriae and lipopolysaccharide (LPS) are essential mediators of this interaction^28^.

In the present study, we report for the first time that *E. coli* K-12 supports the growth of some *Lactobacillus* species during co-culture in minimal medium via a process that requires cell-cell contact or close proximity, but does not require fimbriae or LPS. We consider the significance of this finding in terms of ‘adjacent-possible ecological niches’ in a microbial community.

## Results

**Figure 1:**
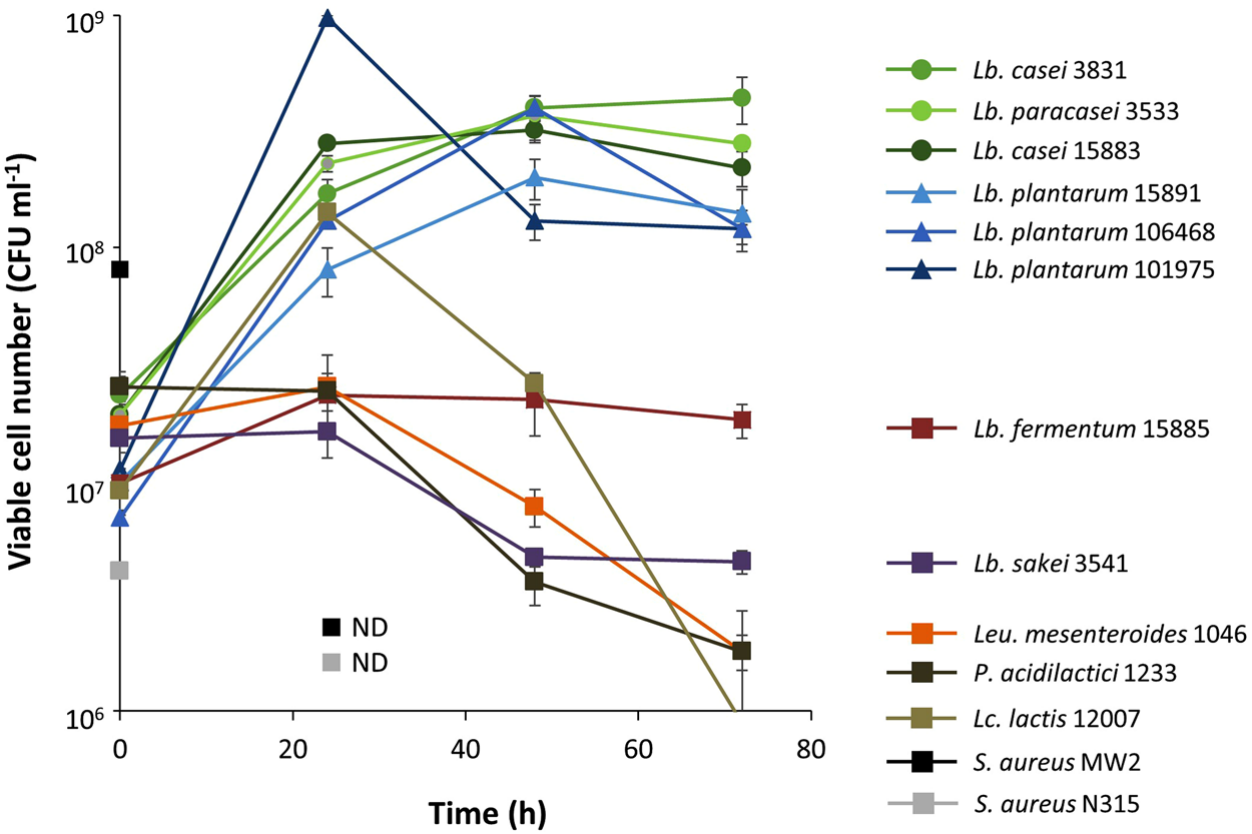
Growth of lactic acid bacteria and *S. aureus* co-cultured with *E. coli*. Each of the pre-cultured strains was inoculated with *E. coli* K-12 BW25113 in M9 medium. The CFU values of *S. aureus* strains were below 10^2^ after 24 h.

Eleven LAB from distinct sources, such as fermented foods, and two *S. aureus* strains, which are partly auxotrophic for amino acids and are close relatives of *Lactococcus* from a genome-wide viewpoint^29^, were tested for their growth in co-culture with *E. coli* K-12. Aside from K-12 itself, none of these strains could grow in mono-culture in M9 minimal medium (Fig. S2). We found that the CFU values of 6 of the tested *Lactobacillus* strains, including 3 belonging to the *L. casei*-group and 3 strains of *L. plantarum*, increased in the presence of *E. coli* (Fig. 1). In contrast, co-culture with *E. coli* did not increase the CFU values of *L. fermentum*, *Leuconostoc mesenteroides*, *Pediococcus acidilactici*, *L. sakei*, or *Lactococcus lactis*. Similarly, *E. coli* did not support the growth of the *S. aureus* strains. Thus, the growth support by *E. coli* was specific for LAB of the *L. casei*-group and *L. plantarum*.

**Figure 2:**
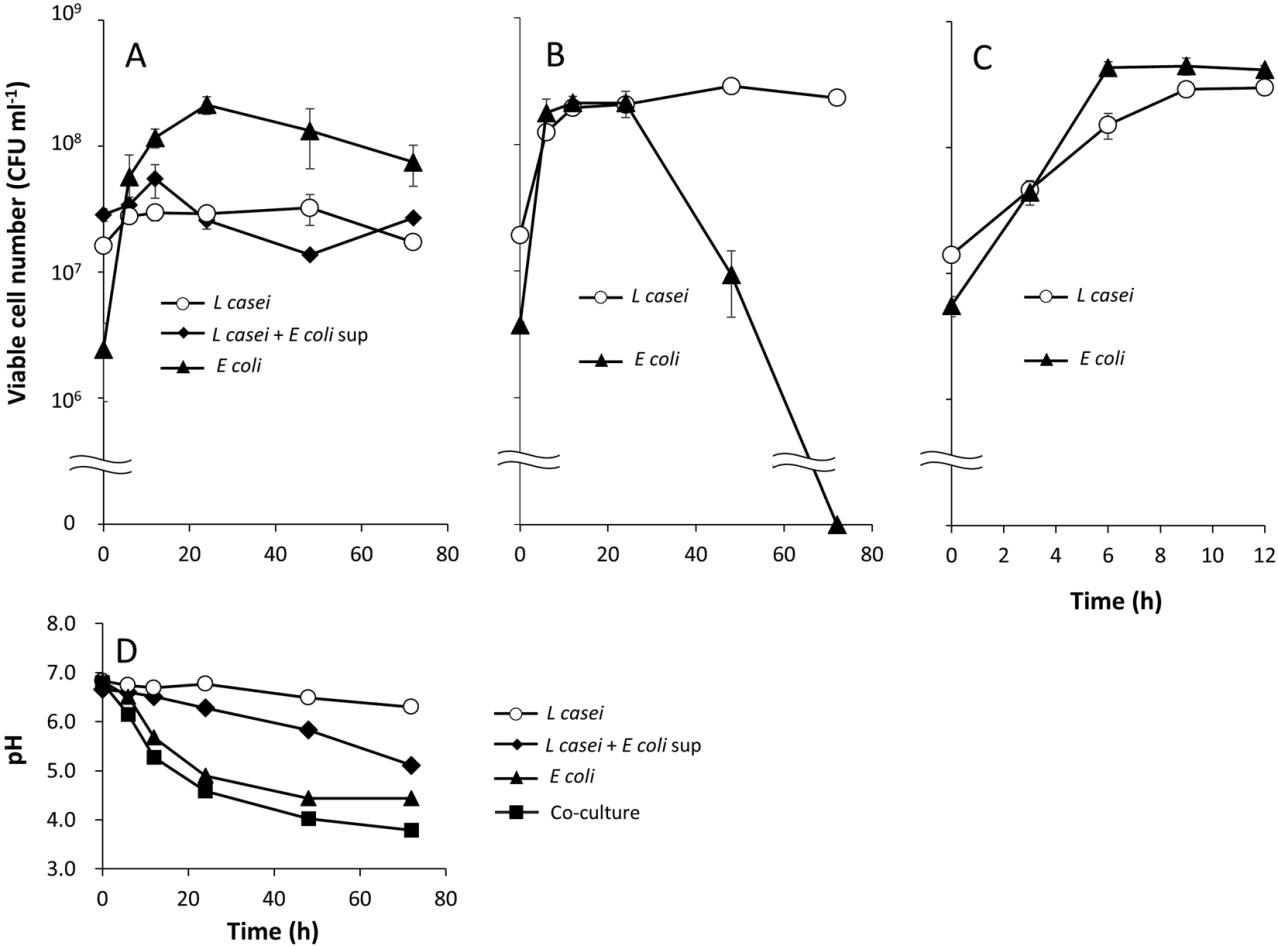
Growth of *L. casei* and *E. coli* during mono-culture and co-culture in M9 medium. (**A**) Mono-cultures of *E. coli*, *L. casei* NBRC 3831, and *L. casei* NBRC 3831 supplemented with *E. coli* culture supernatant (*E. coli* sup). (**B**) Co-culture. (**C**) Co-culture during the first 12 h. (**D**) pH of mono- and co-cultures.

Subsequent co-culture experiments focused on *L. casei* NBRC 3831 as a representative LAB. Mono-culture of *L. casei* in M9 showed little increase in CFU numbers (Fig. 2A), whereas co-culture with *E. coli* supported growth in this medium, with co-cultured *L. casei* reaching a density of 10^8^ CFU ml^−1^ after 6 h (Fig. 2C). During log-phase growth in the co-culture, the specific growth rate was 0.396 h^−1^, approximately 67% that of the mono-culture in MRS (*µ* $\fallingdotseq$ 0.6 h^−1^). After 24 h of co-culturing (Fig. 2B), *L. casei* dominated over *E. coli*.

Next, we monitored carbon consumption and metabolites of these two strains to evaluate metabolic activities during cell proliferation. The concentrations of glucose and of d- and l-lactate in the co-culture were measured (Fig. S3). During mono-culture in M9, *L. casei* consumed glucose and produced l-lactate (despite the lack of increase in CFU number) (Fig. S3A,B). These results showed that *l. casei* is metabolically active even under conditions that are insufficient for cell proliferation, which might provide a useful insight into persistency of health-promoting effects in terms of probiotics. During co-culture, l-lactate production was higher than in the mono-culture, probably due to the increase in CFU number of *L. casei* (Fig. S3B,C). *E. coli*, which is known to be a d-lactate producer, started to produce d-lactate after 10 h of mono-culture in M9; the same pattern was observed in the co-culture in this medium.

**Figure 3:**
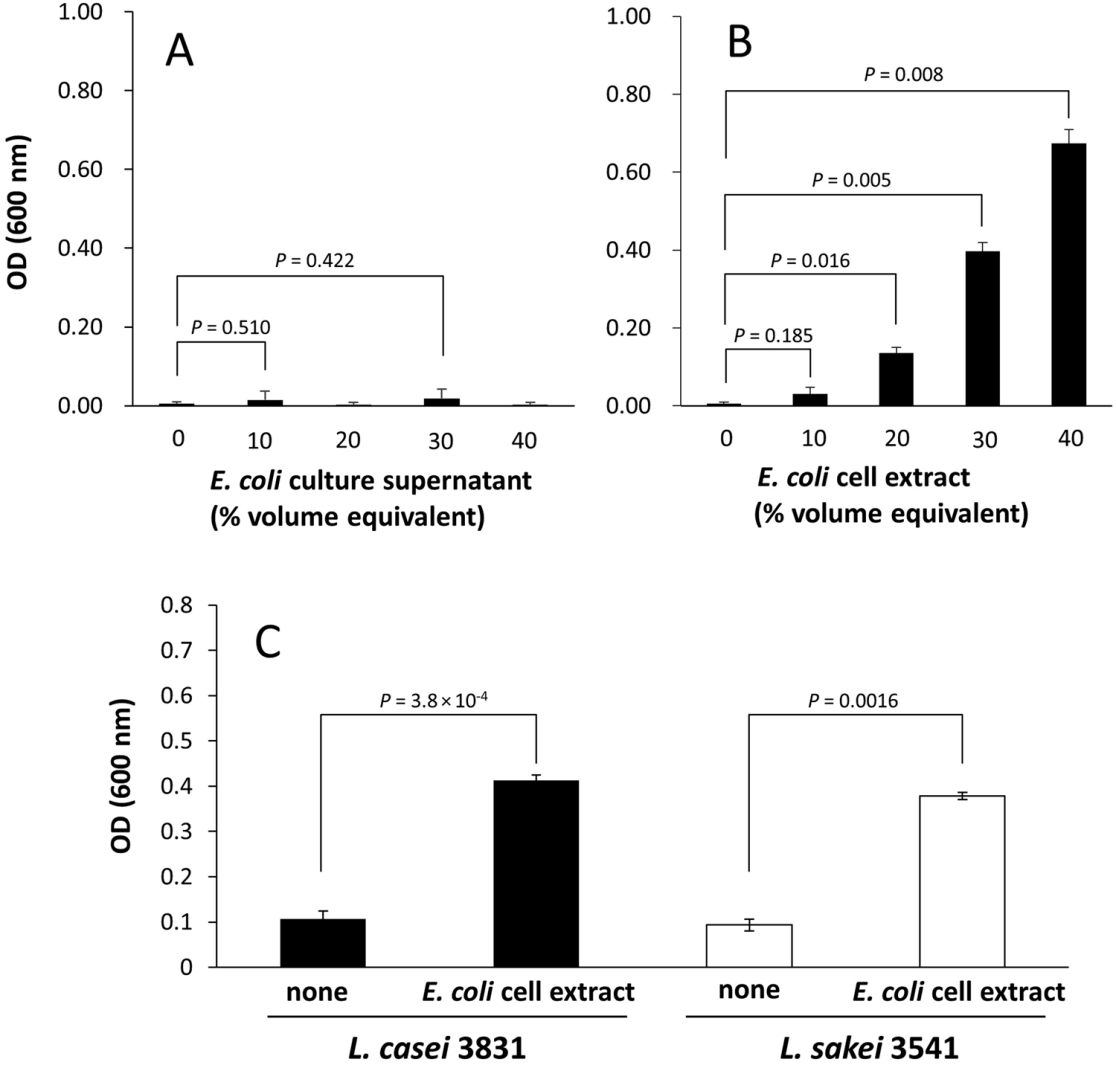
Growth of *L. casei* or *L. sakei* in supplemented M9 media. (**A**) *L. casei* supplemented with culture supernatant from *E. coli* or (**B**) cell-free extract. (**C**) *L. casei* and *L. sakei* in M9 media with and without 30% (vol) *E. coli* cell-free extract. The values in the horizontal axis represent the concentration (vol%) of each supplement. Cultures were grown for 24 h and the optical density was measured. The 40% (vol) of the cell-free extract consists of 6 ml fresh M9 medium and 4 ml cell-free extract in total of 10 ml.

We next tested whether the spent medium or the cell-free extract obtained from mono-culture of *E. coli* in LB would support the growth of *L. casei* in the absence of co-culture (Fig. 3A,B). The spent medium from *E. coli* had no effect when provided at concentrations of 10 to 40% (percent volume equivalent) in M9 medium. These results demonstrated that the growth-supporting factor is not provided by the spent medium obtained from mono-culture of *E. coli* in LB. These observations also excluded the possibility that residual soluble factors carried over from the initial *E. coli* pre-culture supported *L. casei* growth during co-culture. In contrast, cell-free extract at 30% supported the growth of *L. casei* as well as that of *L. sakei* NBRC 3541 (Fig. 3C), a LAB species that was not able to grow when co-cultured with an *E. coli* in minimal medium.

**Figure 4:**
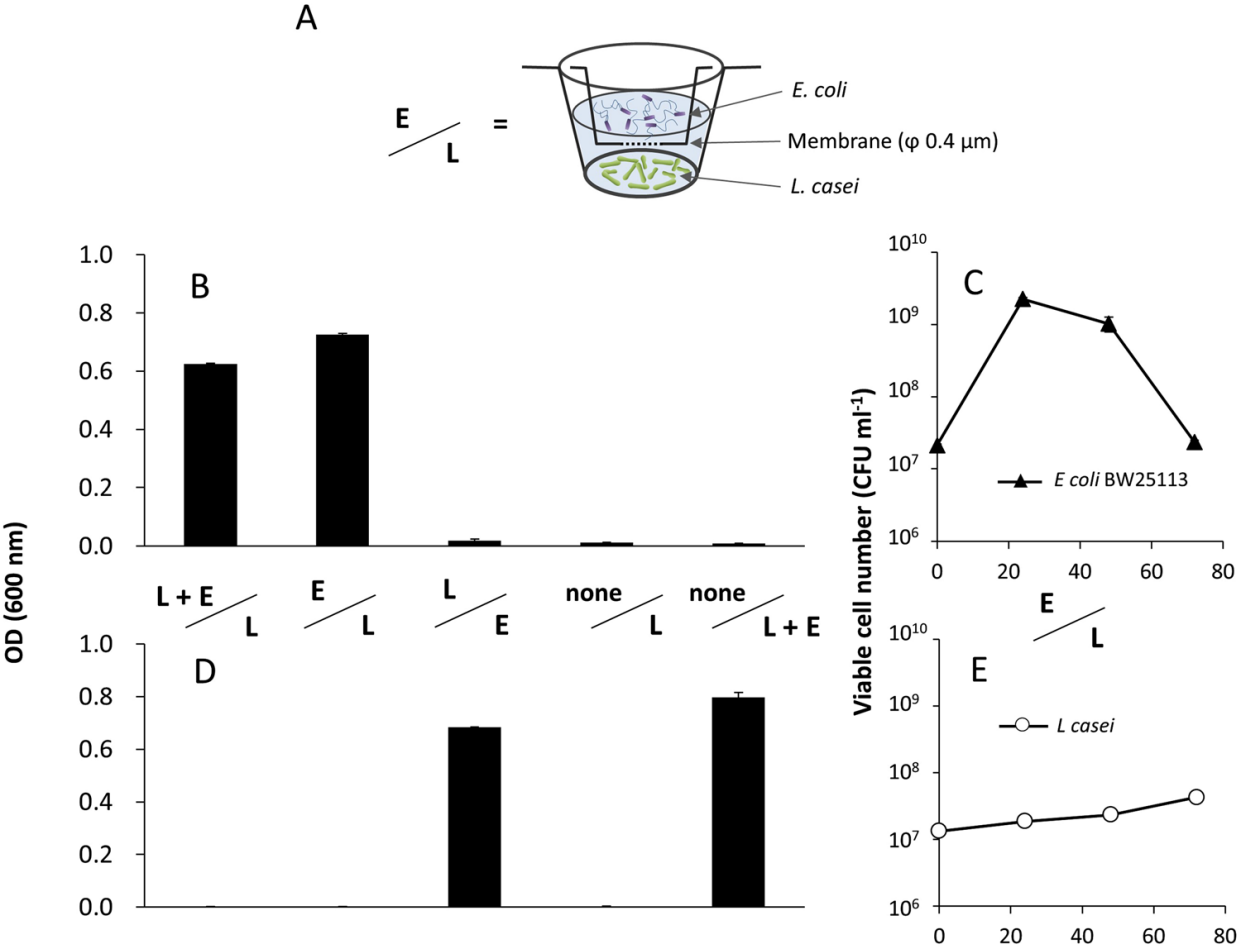
Growth of *L. casei* and *E. coli* in a membrane culture system. (**A**) Schematic diagram of the membrane culture system, where the two compartments were separated by a 0.4-μm membrane. “L”, “E”, and “L + E” represent *L. casei* mono-culture, *E. coli* mono-culture, and co-culture, respectively. (**B**,**C**) Upper side of the membrane. (**D**,**E**) Bottom side. (**B**,**D**) Optical density after 24 h. (**C**,**E**) CFU values for 72 h.

Since the *E. coli* spent medium was insufficient to support the growth of *L. casei* in M9, we hypothesized that growth effects on *L. casei* require close proximity with *E. coli* cells. To test this theory, we used a membrane culture system wherein *L. casei* and *E. coli* were separated by a membrane (pore size, 0.4 µm). No growth support was observed for *L. casei* placed in M9 in a compartment adjacent to a parallel *E. coli* culture. Addition of *L. casei* to the *E. coli* compartment did not affect this result (Fig. 4, L+E vs. L). Thus, direct interaction with, or close proximity to, *E. coli* cells is required to permit the observed growth-supporting effect on *L. casei* growing in minimal medium (Fig. 4).

**Figure 5:**
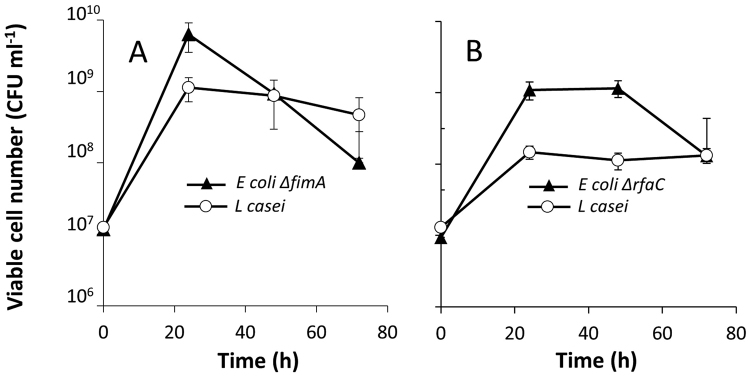
Growth of co-cultured *L. casei* NBRC 3831 with (**A**) *E. coli ΔfimA*, (**B**) *E. coli ΔrfaC*.

As noted above, we previously showed that fimbriae and LPS are essential mediators of some co-aggregation processes^28^. We postulated that the same might apply to the co-culture phenomenon described in the present work. Therefore, we evaluated the growth-supporting effect of *E. coli* using strains deleted for either *fimA* (the structural gene of the type I fimbriae^30^) or *rfaC* (yielding a deep-rough LPS mutant with a truncated core LPS oligosaccharide^31^). Notably, both of these *E. coli* mutants were able to support the growth of *L. casei* upon co-culture (Fig. 5), demonstrating that the contact/proximity-based co-culture effect observed in the present work does not depend on fimbriae or LPS.

**Figure 6:**
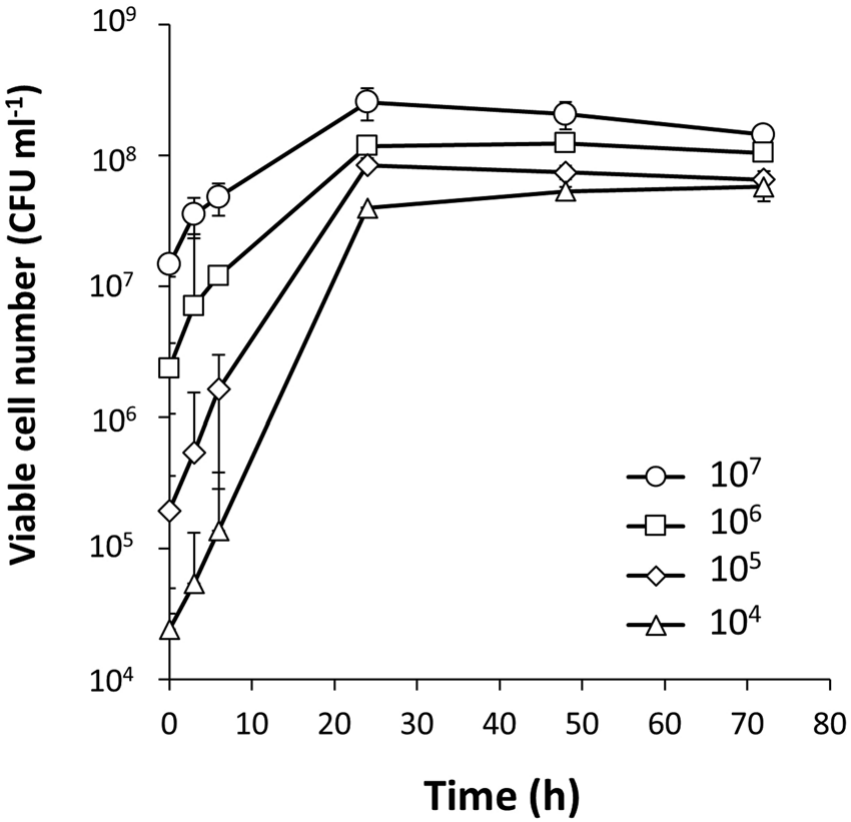
Effect of the initial inoculum number of *L. casei* in co-culture with *E. coli*. The initial inoculum numbers of *E. coli* are 10^6^ cells ml^−1^. The specific growth rates (*μ*) of *L. casei* (3–6 h) inoculated from the initial cell numbers 10^4^, 10^5^, 10^6^, and 10^7^ were 0.311, 0.370, 0.177, 0.102, and 0.302 h^−1^, respectively.

In the above co-culture experiments, the initial inoculum ratio was 10:1 (*L. casei*: *E. coli*). We wanted to test if the growth support could be observed even when *L. casei* existed as a minority component of the co-culture. As shown in Fig. 6, growth was robust even when the culture was initiated using *L. casei* at 10^4^ CFU ml^−1^, that is, at an initial inoculum ratio of 1:100 (*L. casei*: *E. coli*). This result suggested that the retrieval of the growth-supporting factor(s) from *E. coli* does not rely on having high initial density of LAB, in contrast to other systems, for instance, as in the induction of LAB bacteriocins by quorum sensing^32^.

**Figure 7:**
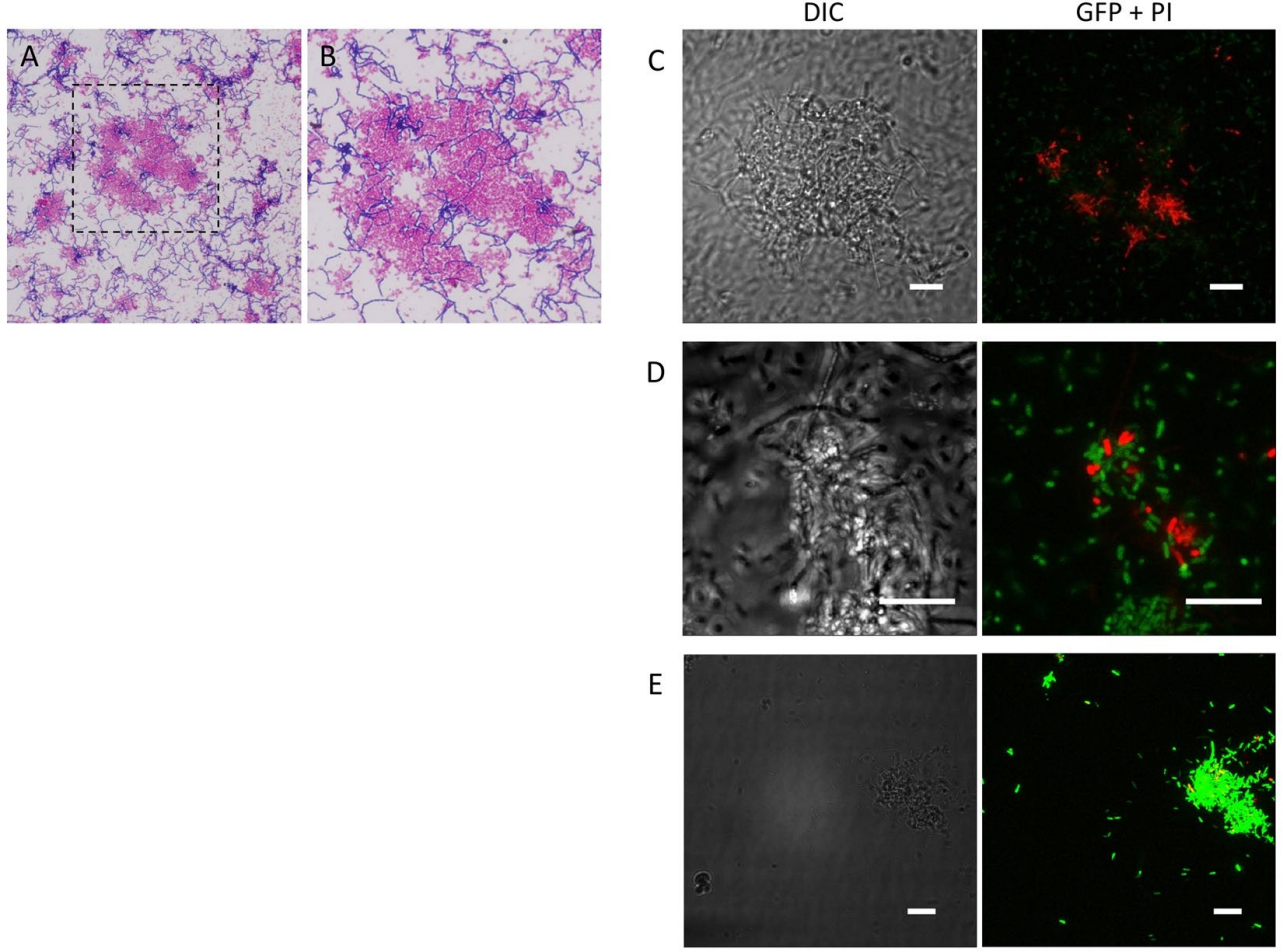
Microscopic analysis of micro-scale clusters from a 3-h co-culture of *L. casei* NBRC 3831 and *E. coli*. (**A**–**D**) *L. casei* NBRC 3831 and *E. coli* (BW25113-GFP) co-culture. (**E**) *E. coli* mono-culture. (**A**,**B**) are Gram-stained images. The box in (**A**) shows the region magnified in (**B**). (**C**–**E**) DIC and merged GFP and PI pictures. GFP and PI images are overlaid confocal images of 15–30 slices, 5–25 µm depths. Bars = 10 µm.

We used Gram staining to observe cells after 3 h of co-culturing (Fig. 7A,B). The *L. casei* cells formed long chains. Individual *L. casei* cells were observed embedded in clusters of *E. coli* cells. To exclude a possible artifact during the fixation and Gram-staining procedure, an unfixed sample from 3-h co-culture of *L. casei* and *E. coli* was directly observed by fluorescence microscopy. In addition to the free *E. coli* cells (expressing GFP), clusters carrying embedded *L. casei* cells were observed (compare Fig. 7D). The viability of cells was analyzed by PI staining (Fig. 7C–E). PI is an intercalating DNA stain that penetrates injured membranes; thus, PI staining is indicative of dead cells. Notably, cells located within micro-scale clusters were often positive for PI staining (Fig. 7C,D) and were presumably dead. In *E. coli* mono-cultures, PI-positive cells were rare, though not undetectable (Figs 7E and S4). Considered together, these results indicate that *L. casei* has the ability to interact with *E. coli* cells and form micro-scale clusters accumulating PI-positive cells, which could explain how *L. casei* uptakes certain growth-supporting factors from *E. coli* during co-culture.

## Discussion

This study demonstrated for the first time that LAB, which are strictly auxotrophic, can interact with co-cultured *E. coli* to proliferate in what would otherwise be nutrient-limiting conditions. Such growth ability was found to be specific for some *Lactobacillus* species, but the reason for the observed specificity remains to be clarified. This specificity is not simply attributable to the diversity of the nutrient auxotrophies among LAB species, because non-co-culturable species (such as *L. sakei*) were able to grow when cultured on minimal medium supplemented with an *E. coli* cell extract. It is also unlikely that the observed growth results from genetic reversion of auxotrophy by mutation^33^, given that a starting inoculum as small as 10^4^ CFU ml^−1^ yielded a swift growth during co-culture. Secreted molecules such as antimicrobial peptides and chemoattractants would be among the candidates responsible for the observed strain specificity, however the spent medium of a mono-culture of *L. casei* NBRC 3831 in MRS did not induce PI-positive *E. coli* cells (Fig. S5). In this regard, it might be important to note that *E. coli* tended to assemble around *L. casei* NBRC 3831 in co-culture. For instance, CO_2_, necessary for growth of lactobacilli^34^, might be associated with the cell-cell contact or close proximity. It might be also important to note that growth simulation analysis of a co-culture of LAB and *E. coli* predicted that some *E. coli*-derived factors could improve LAB growth, although it was in a condition in which LAB can grow in the absence of *E. coli* ^35^.

Macro-scale co-aggregation requires acidic conditions (below pH 5.0)^28^, whereas the pH in the exponential growth phase of the co-culture in this study was above 6.0 (Fig. 2D). This result suggested that the co-clusters observed in the present study are based on a mechanism distinct from the fimbria- or LPS-mediated mechanism used for macro-scale co-aggregation. Thus, we propose that the growth support observed concomitantly with the formation of micro-scale co-clusters relies on a mechanism distinct from the previously reported macro-scale co-aggregation.

It was not elucidated in the present study whether the micro-scale co-cluster itself is essential for the growth support. However, it is a new finding that *L. casei* can proliferate only when this LAB is provided with cell-cell contact or close proximity to *E. coli*, providing us a new ecological viewpoint of how an auxotrophic minority in a microbiota can exploit its ecological niche. Here, we would term such an ecological concept as an ‘adjacent-possible ecological niche (APEN)’. The ‘adjacent-possible’ concept itself was previously proposed by the theoretical biologist Stuart Kauffman not only in the context of biology but also in a broad range of scientific fields, including economics^36,37^. Considering LAB as the minority (<0.1%) in the dense colonic microbiota (10^10^–10^12^ cells/g)^38^ as well as in the microbiome of fermented food, an ecological viewpoint of such an adjacency would have relevance in the context of LAB physiology. The better understanding of APEN may permit us to expand our scope beyond the one currently limited to a narrow set of interactions between LAB and yeasts^24^ or cheese-isolated *E. coli* ^35^ and between *L. bulgaricus* and *Streptococcus thermophilus* ^39^. The species specificities and overall relevance of APENs to the LAB lifestyle, as well as the bacterial strategies targeting APENs, are intriguing questions that will need be addressed in future studies.

In conclusion, *L. casei* group and *L. plantarum* strains became culturable in M9 medium when co-cultured with *E. coli* K-12. This growth support did not reflect “carry-over” of soluble nutrients from the pre-culture supernatant or the leakage of compounds from the mono-cultured *E. coli* cells. The growth of *L. casei* NBRC 3831 required cell-cell contact or close proximity to *E. coli* cells, suggesting that *L. casei* NBRC 3831 uptakes growth-supporting factor(s) from nearby *E. coli* cells.

## Methods

### Strains and culture conditions

The strains used in this study are listed in Table 1. The parent wild-type strain *E. coli* K-12 and the deletion strains Δ*fimA* (JW4277) and Δ*rfaC* (JW3596) of the KEIO collection were obtained from the National Institute of Genetics (Shizuoka, JAPAN). *Lactobacillus* strains were statically cultured in 10 ml of de Man, Rogosa, and Sharpe (MRS) medium (Oxoid, Hampshire, England) (10 g proteose peptone, 10 g beef extract, 5 g yeast extract, 20 g glucose, 1 g polysorbate, 2 g ammonium citrate, 5 g sodium acetate, 0.1 g magnesium sulfate, 0.05 g manganese sulfate, and 2 g dipotassium phosphate per liter of deionized water) for 24 h at 37 °C. *E. coli* was cultured in 10 ml of lysogeny (Luria) broth (LB) medium (5 g yeast extract, 10 g peptone, and 10 g NaCl per liter of deionized water) in a reciprocal shaker at 180 rpm for 18–20 h at 37 °C. Two laboratory model strains of *Staphylococcus aureus*, MW2 and N315, were cultured in Brain Heart Infusion (BHI) medium (Becton Dickinson, New Jersey, USA) on a reciprocal shaker at 180 rpm for 18–20 h at 37 °C.

**Table 1:** Strains used in this study.

Strain	Description	Reference
***Escherichia coli***		
BW25113	Parent strain of the mutants; ∆*(araD-araB)567*, ∆*lacZ4787*(::rrnB-3), lambda^−^, *rph-1*, ∆*(rhaD-rhaB)568*, *hsdR514*	45
JW3596	LPS deletion mutant; *ΔrfaC*	46
JW4277	Type I fimbriae deletion mutant; *ΔfimA*	46
BW25113-GFP	BW25113 carrying pRIT-P*ldh-gfp*	This study
***Staphylococcus aureus***		
MW2	Methicillin-resistant community-acquired isolate	47
N315	Methicillin-resistant clinical isolate	29
**Co-culturable LAB**		
*Lactobacillus casei* subsp. *rhamnosus* NBRC 3831	Isolated from the brewing process of Japanese sake	
*Lactobacillus paracasei* subsp. *paracasei* NBRC 3533	“Hiochi bacterium” in Japanese sake	48
*Lactobacillus casei* NBRC 15883	Isolated from cheese	
*Lactobacillus plantarum* NBRC 15891	Isolated from pickled cabbage	
*Lactobacillus plantarum* NBRC 106468	Isolated from fermented cassava roots	
*Lactobacillus plantarum* NBRC 101975	Isolated from non-salted fermented vegetable	
**Non co-culturable LAB**		
*Leuconostoc mesenteroides* IAM 1046	Dextran producer	
*Pediococcus acidilactici* NBRC 3076		
*Lactococcus lactis* subsp. *lactis* NBRC 12007	Bacteriocin producer	
*Lactobacillus sakei* NBRC 3541	Isolated from sake-moto	
*Lactobacillus fermentum* NBRC 15885	Isolated from fermented beets	

### Construction of pRIT-*Pldh-gfp* and recombinant strains

The promoter region of the *Listeria monocytogenes ldh* (lactate dehydrogenase) gene was amplified with primers V25 (5′-TAAGTCGACTTTCTTGCCGTCCACAG-3′) and V19 (5′-CTAGTCGACTTCGAATTCCTCCTA-3′) from the genome of *Listeria monocytogenes* EGDe (http://genolist.pasteur.fr/ListiList/index.html). The resulting fragment was digested with *Sal*I and then cloned into the *Sal*I site of the pRIT-*gfp* plasmid^40^. The resulting plasmid, designated pRIT-*Pldh-gfp*, was transformed into *E. coli* K-12 BW25113 and JW4277 using a standard electroporation protocol^41^.

### Co-culture conditions

Strains were individually pre-cultured as described above, and inoculated (at 1:100) alone or together into M9 medium (12.8 g Na_2_HPO_4_·7H_2_O, 3 g KH_2_PO_4_, 0.5 g NaCl, and 1 g NH_4_Cl per liter of deionized water containing 2 mM MgSO_4_ and 0.1 mM CaCl_2_) supplemented with 2% glucose. For the time course analysis, 10 ml co-culture was statically cultured for 72 h at 37 °C. The necessity of cell-cell contact was tested in a 6-well Millicell^®^ plate (Millipore, Massachusetts, USA) with polyethylene terephthalate membranes (pore size, 0.4 µm) to prevent direct cell-cell contact between the two species. The total volume of a well consisted of 5 ml M9 medium, of which the upper and lower compartments held about 2 and 3 ml of the medium, respectively. The individual pre-cultures were inoculated at 1:100 into the respective compartments. The 6-well plate then was statically cultured at 37 °C. Growth was monitored by optical density (600 nm) and viable cell counts (CFU ml^−1^).

To determine the viable cell number, serial dilutions of the cultures were inoculated onto agar plates, and the CFUs (colony-forming units) were determined. CFU values are presented as the means ± standard errors of 3 independent experiments.

In order to determine the viable cell number from co-culture, we used LB agar medium for *E. coli* and pH modified MRS agar medium to specifically detect *Lactobacillus* colonies. The pH of the MRS agar was adjusted to 6.0 with acetate, which has an inhibitory effect on *E. coli* growth^42^. The CFU values of *Lactobacillus* species were comparable between normal MRS agar and acetate-MRS agar (Fig. S1A). The colonies on the acetate MRS agar were confirmed as *Lactobacillus* by sequencing the PCR-amplified 16 S rRNA genes with primers 341 F (5′-CCTACGGGAGGCAGCAG-3′) and 907 R (5′-CCGTCAATT CCTTT[A/G]AGTTT-3′)^43^ (Fig. S1B).

### Evaluation of the specific growth rate of *L. casei* NBRC 3831

The specific growth rate (*μ*) in the mid-exponential growth phase (3–6 h) of *L. casei* NBRC 3831 in MRS mono-culture and M9 co-culture was calculated by the following equation:

$$ln{X}_{t}=ln{X}_{t0}+\mu t$$

where *X*_*t*_ is the CFU value at hour *t*, and *X*_*t0*_ is the initial CFU value.

### Preparation of spent medium and cell-free extract from *E. coli* pre-culture

The cells and spent medium from a 10 ml *E. coli* pre-culture were separated by centrifugation at 1,500 g for 20 min at 4 °C. The supernatant was filter sterilized (0.2-µm cellulose membrane) and used for further analysis (“spent medium”). The collected cells were washed 3 times with phosphate-buffered saline (PBS, pH 7.0), re-suspended in 1.5 ml of PBS, and disrupted with sterile glass beads (diameter, 0.1 mm) using a cell disruptor (Disruptor Genie, Scientific Industries, NY, USA): a 2-ml tube containing 1.4 g beads and the cell suspension was mixed for 20 min with intervals to cool the tube in an ice-water bath. Following disruption, the volume of the mixture was raised to a total volume of 10 ml using PBS and centrifuged at 10,000 g for 20 min at 4 °C. The resulting supernatant was filtrated (0.2-µm cellulose membrane) and used as a cell-free extract for further analysis.

### Microscopic analysis

Gram staining was conducted using a commercial kit (Nissui Co., Tokyo, Japan); the stained samples were observed using a FSX100 inverted microscope (Olympus, Tokyo, Japan). Cell viability in a co-aggregate was evaluated in a 3- to 5-h co-culture of *L. casei* and GFP-expressing *E. coli*. The cells were stained with 3.5 µM propidium iodide (PI)^44^. Fluorescence microscopy was performed using a Leica TCS SP5 confocal laser microscopic system (Leica Microsystem Co., Wetzlar, Germany). The excitation wavelengths used were: 395 nm for GFP and 488 nm for PI. The emission was observed at 509 nm and 633 nm, respectively. Images were acquired and analyzed using Leica LAS AF software, version 2.6.0.

### Determination of glucose and lactate concentrations

Glucose and lactic acids concentrations were measured using commercial kits according to the manufacturer’s instructions. Glucose consumption was determined by the Glucose Test Kit Wako (Wako Chemical Co. Japan), and the production of l-(+)- and d-(−)-lactic acids was measured by a colorimetric method using the F-kit (Roche, Basel, Switzerland).

### Statistical Analysis

All experiments were conducted in triplicate. Values represent the mean ± SEM of three independent experiments. To compare two groups, student’s test was used and P < 0.05 was considered as statistically significant.

## Electronic supplementary material

Supplementary Information
