# Supplementary material for: Adjacent-possible ecological niche: growth of Lactobacillus species co-cultured with Escherichia coli in a synthetic minimal medium
Source: Sci Rep. 2017 Oct 16;7:12880. doi: 10.1038/s41598-017-12894-3 (PMC5643319; doi:10.1038/s41598-017-12894-3)
Supplement: Supplementary file 1 — Supplementary Information [file 41598_2017_12894_MOESM1_ESM.pdf]

Supplementary Information

Adjacent-possible ecological niche: growth of *Lactobacillus* species co-cultured with *Escherichia coli* in a synthetic minimal medium

Kouhei Mizuno[1]*, Mamiko Mizuno[2], Mio Yamauchi[1], Aya J. Takemura[3], Veronica Medrano Romero[2], Kazuya Morikawa[4]*

[1]*Department of Creative Engineering, National Institute of Technology, Kitakyushu College, Kitakyushu 802-0985, Japan*

[2]*Graduate School of Comprehensive Human Sciences, University of Tsukuba, Tsukuba, 305-8575, Japan*

[3]*PhD Program in Human Biology, School of Integrative and Global Majors, University of Tsukuba, Tsukuba, 305-8575, Japan*

[4]*Division of Biomedical Science, Faculty of Medicine, University of Tsukuba, Tsukuba 305-8575, Japan*

*Corresponding Author: Dr. Kouhei Mizuno and Dr. Kazuya Morikawa

E-mail: mizuno@kct.ac.jp (K. Mizuno), Tel/Fax: +81-93-964-7303 (K. Mizuno)

E-mail: morikawa.kazuya.ga@u.tsukuba.ac.jp (K. Morikawa), Tel/Fax: +81-29-853-3928 (K. Morikawa)

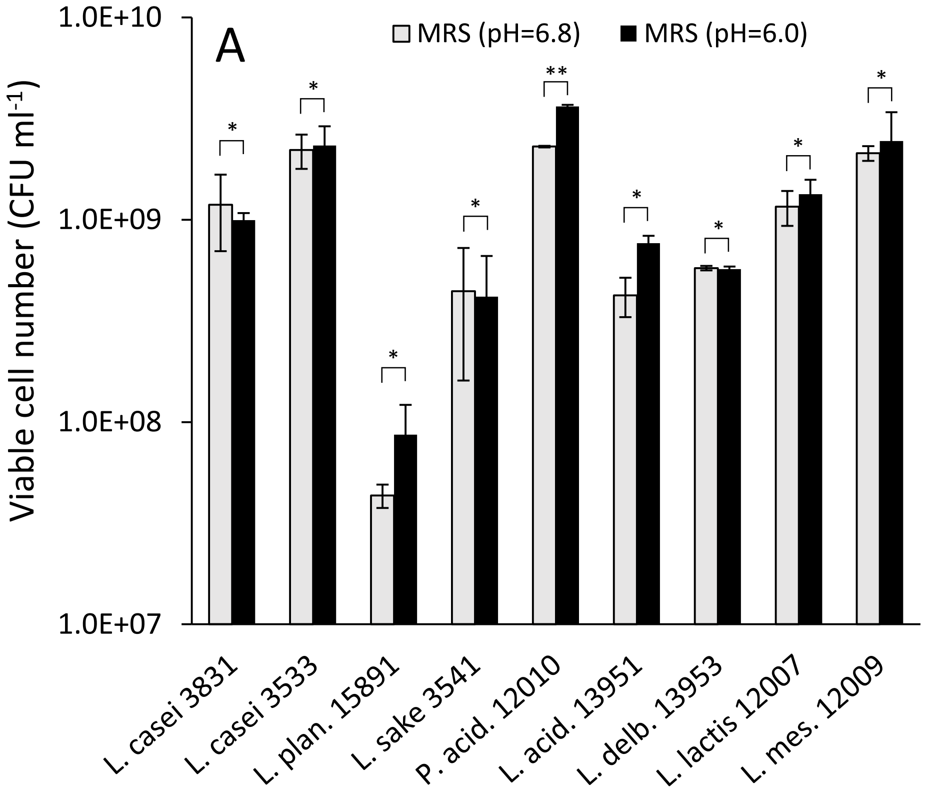

* P > 0.05, ** p < 0.05

B

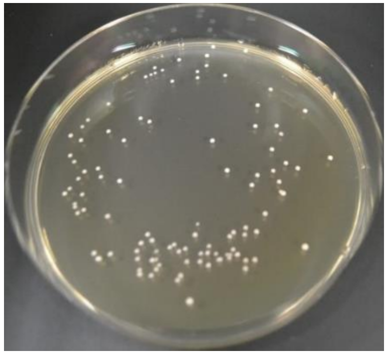

L. casei colonies on an acetate-MRS plate.

Genetic identification of colonies on an acetate-MRS plate (pH=6.0) from a co-culture

Colony number	Closest match of 16S rRNA	Genome ID	Identity (%)
67	*L. casei* ATCC 334	NC_008526.1	100
1	*L. casei* ATCC 334	NC_008526.1	99
Total 68			

Figure S1. Confirmation of the specific detection of LAB on MRS plates with pH adjusted to 6.0 by acetate.

(A) Colony forming units were tested for representative LAB on MRS-agar pH 6.0 or MRS-agar pH6.8 plates after 48 h incubation. (B) Colonies from co-culture (*L. casei* NBRC 3831 and *E. coli*) on an acetate-MRS plate and their identification by direct-sequencing of 16S rRNA genes.

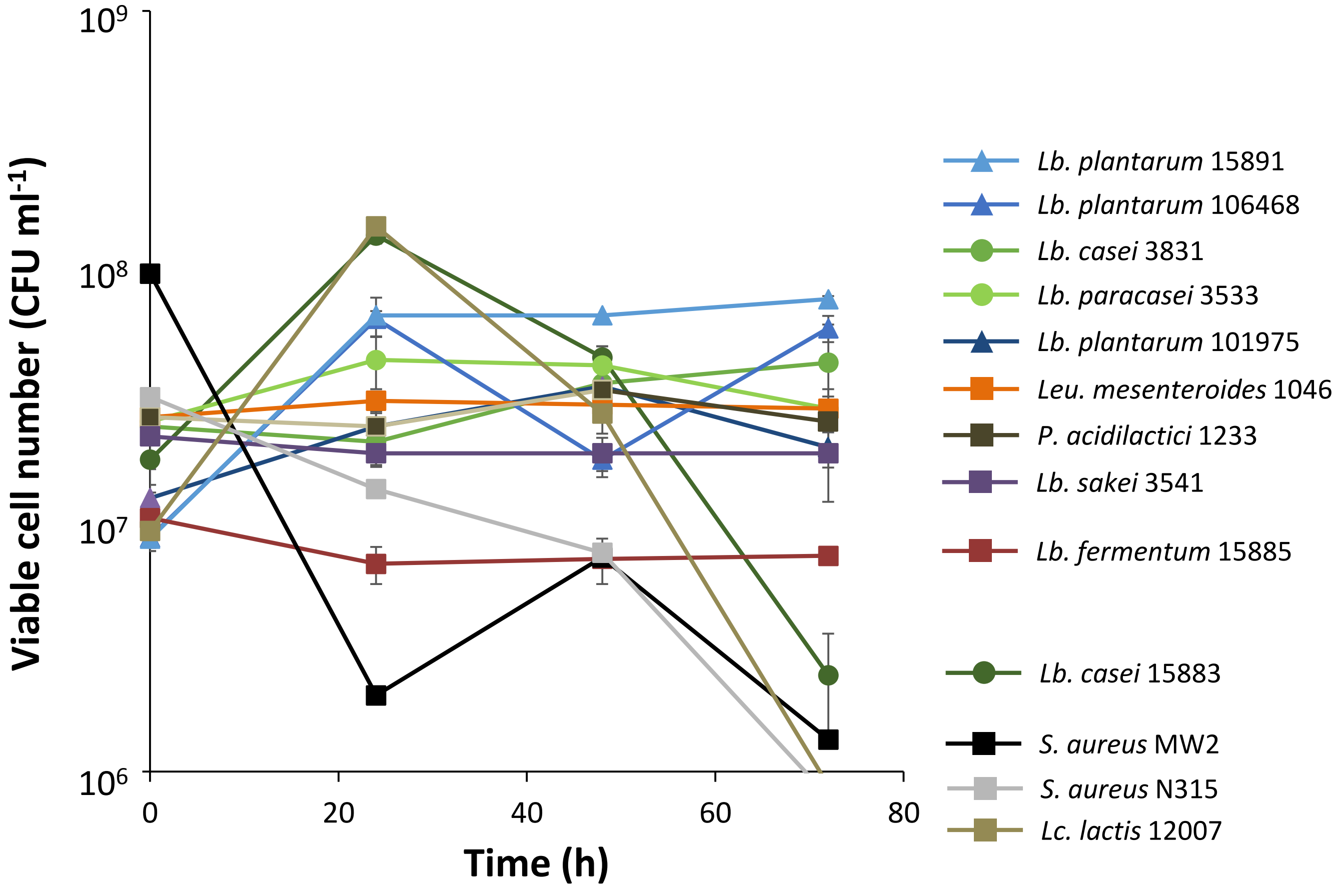

Figure S2. Growth of lactic acid bacteria and *S. aureus* mono-cultures in M9 medium.

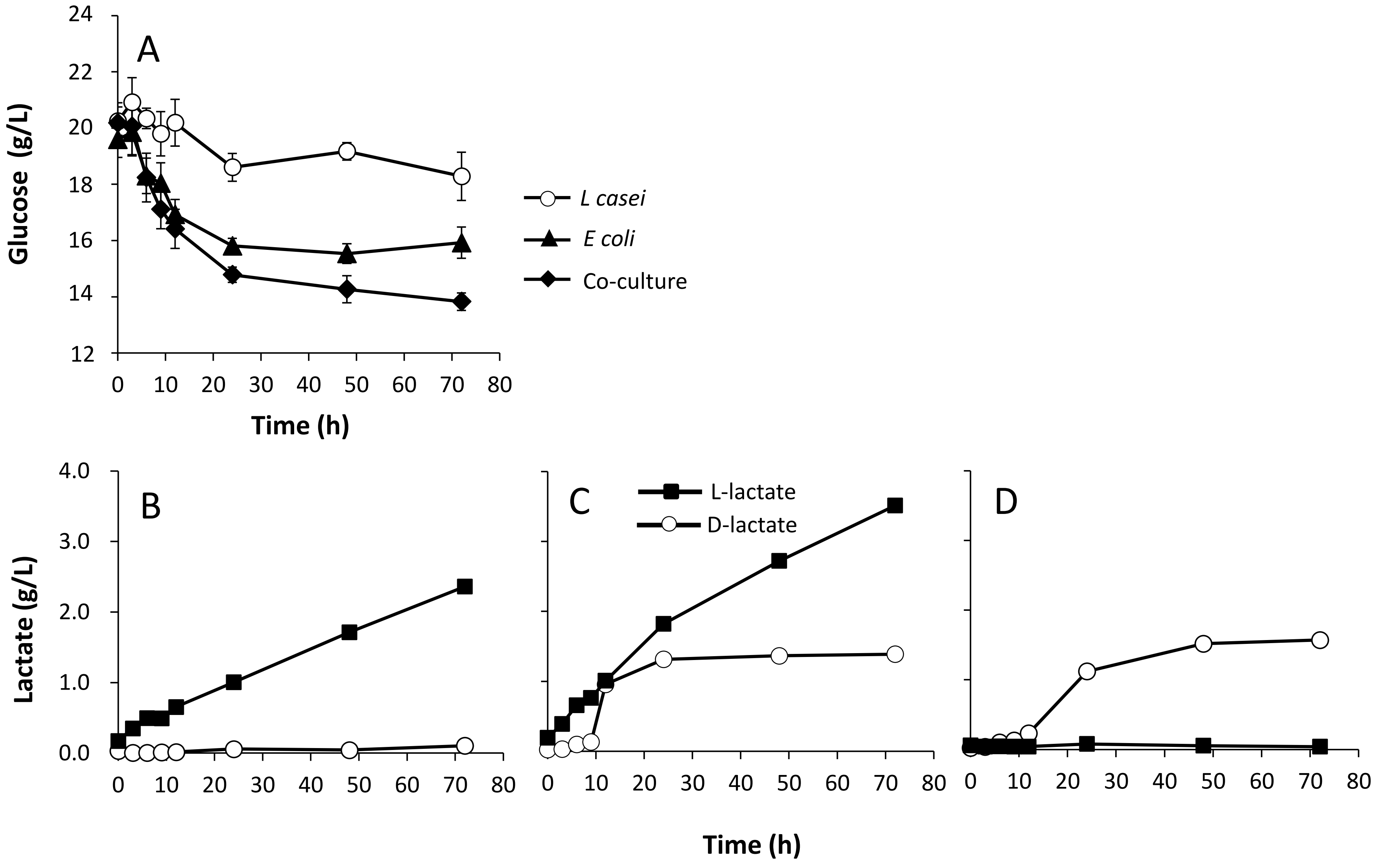

Figure S3. Glucose consumption and lactate production in mono- and co-cultures.

(A) Glucose concentration. (B)–(D) Lactate production. (B) *L. casei* NBRC 3831 mono-culture, (C) Co-culture, (D) *E. coli* mono-culture.

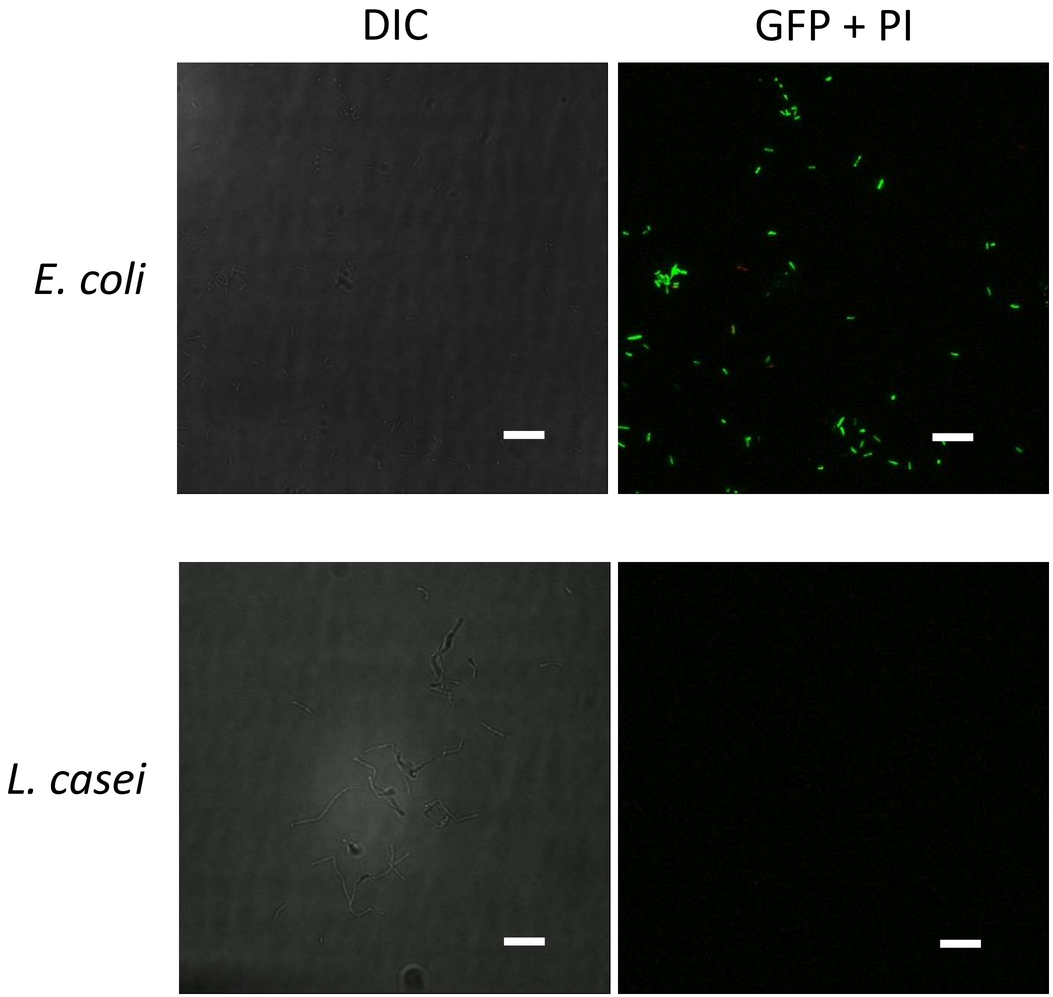

Figure S4. Representative microscopic images of 3-h mono-cultures of *E. coli* (BW25113-GFP) and *L. casei* NBRC 3831. GFP and PI images are overlaid confocal images of 16–22 slices, 16–22 μm depths. Bars = 10 μm.

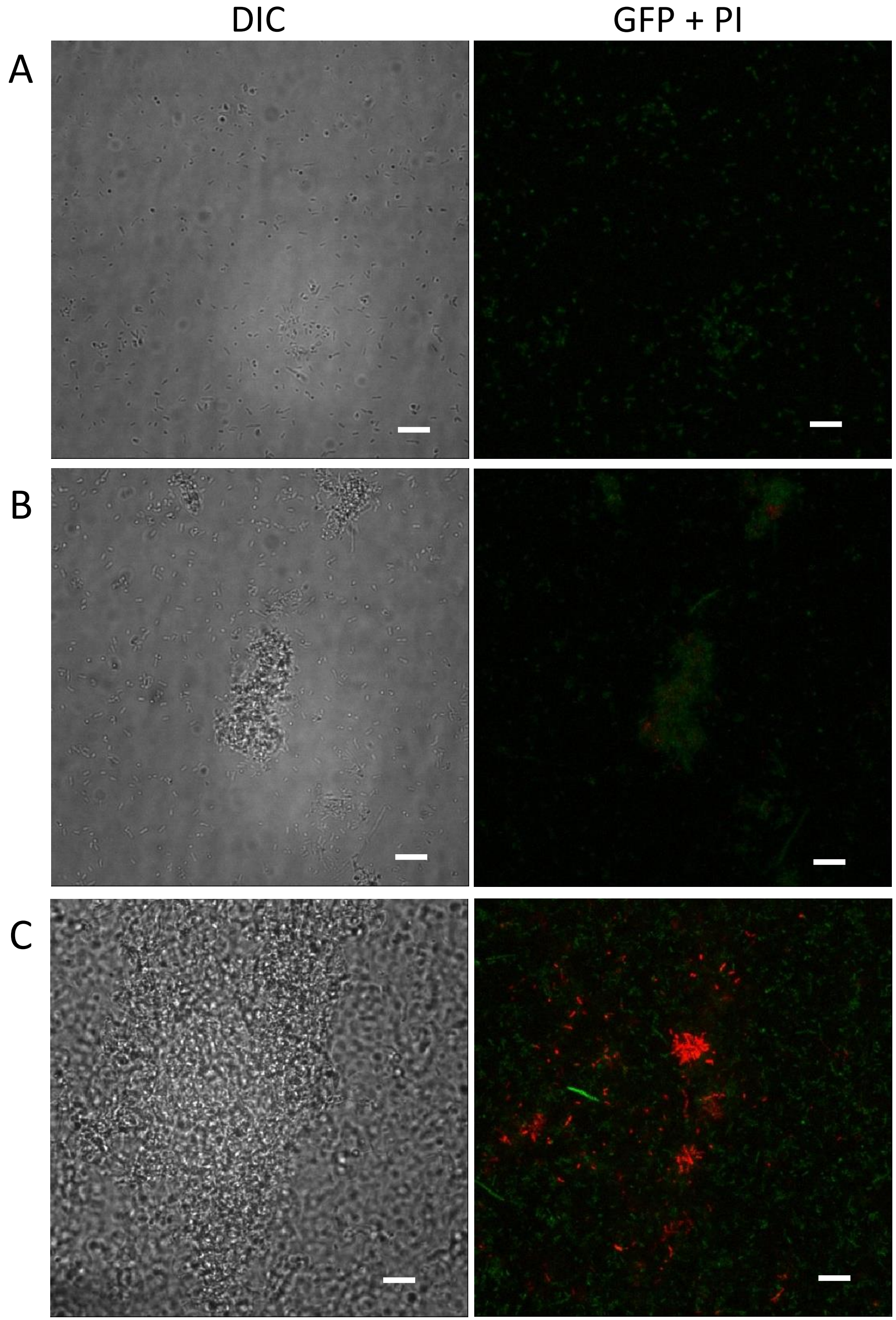

Figure S5. Effect of *L. casei* supernatant on cell viability of *E. coli* mono-cultures.

(A) and (B) *E. coli* mono-cultures were supplemented with 10% volume of the supernatant from *L. casei* overnight culture and incubated for 3 h. (C) Co-culture image (3 h) showing PI-positive cells obtained in the concurrent experiment. GFP and PI images are overlaid confocal images of 22–46 slices, 15–45 μm depth. Bars = 10 μm.

No significant PI signal was observed in the *E. coli* mono-cultures supplemented with the *L. casei* supernatant (overnight culture), while PI positive cells accumulated in the co-culture system.
